# Greater Happiness for a Greater Number: Did the Promise of Enlightenment Come True?

**DOI:** 10.1007/s11205-015-1128-x

**Published:** 2015-10-19

**Authors:** Ruut Veenhoven

**Affiliations:** 1 Erasmus Happiness Economics Research Organization, Erasmus University Rotterdam, Rotterdam, The Netherlands; 2 Optentia Research Program, North-West University, Vanderbijlpark, South Africa

**Keywords:** Happiness, Life satisfaction, Subjective well-being, Trend analysis

## Abstract

In the eighteenth century ‘Enlightened’ thinkers challenged the belief that happiness exists only in Heaven. They claimed that happiness is possible in earthly life and foresaw that greater happiness would be achieved using reason. Did this promise of greater happiness come true? Several scholars doubt that we have become any happier and some claim that happiness has declined. These critical claims are tested using the time trend data available in the World Database of Happiness, which cover the period 1950–2010 and involve 1531 data points in 67 nations yielding 199 time-series ranging for 10 to more than 40 years. The analysis reveals that happiness has risen in most nations. The average yearly rise in the 67 nations was +0.012 on scale 0–10, which equals a rise of one full point every 83 years. At this rate happiness must have improved by more than two points over the past two centuries and, together with increasing longevity, this denotes an unprecedented rise in happy life years.

## Introduction

### Views on Average Happiness

Thinking about happiness was not very positive in Europe in the ‘dark’ middle ages. Thought was dominated by the church, which rather glorified suffering, with the crucified Christ as its main symbol. In the religious perspective happiness had existed in Paradise before the fall and would be bestowed on true believers in afterlife, but was not to be found in earthly life. Earthly happiness was not only deemed to be impossible, but also undesirable. God had not expelled us from Paradise to enjoy life, we were born in sin and suffering was seen as a way to clean our souls from sin and thus to prepare for entrance to Heaven. Though not all church fathers denounced earthly happiness equally much, this pessimistic view prevailed, among other things because life was typically short and brutish in this phase of societal development (Maryanski and Turner 1992; Sanderson 1995).

### Promise of Greater Happiness

The intellectual ‘Enlightenment’, which began in the seventeenth century in Europe, involved two radical changes in thinking: an orientation on facts rather than on religious revelation and the use of reason rather than following custom and belief. In this context a different view on happiness emerged. Happiness came to be seen as something that is possible on earth, which we should not renounce, and that greater happiness can be achieved with the use of reason. Happiness also came to be seen as something desirable, which deserves to be promoted.

A lively discussion emerged on what happiness is precisely, how it can be promoted and whether the state should care about the happiness of its citizens. These matters were discussed in many newly emerging scientific societies and propagated in numerous books and pamphlets. The literature in France is summarized in the monumental book by Mauzi (1960) and the discussion in the Netherlands is aptly described in Buijs (2007).

A radical variant of enlightened thought on happiness developed in the late eighteenth century in Scotland and is described by Bentham (1789) in his famous book *On Morals and Legislation*. Happiness is defined as subjective enjoyment of life, the sum of pleasures and pains, and proclaimed to be the basis of morality. The good or bad of all action should be judged by its effects on happiness, the morally best alternative being the one that yields the ‘greatest happiness of the greatest number’ of people. This consequential ethic is known as ‘utilitarianism’. Applied to policy making it means that governments should aim to promote their citizen’s happiness in the first place and should do so by following fact and reason rather than ideology.

What ever it’s precise definition or moral appreciation, the idea of happiness became part of the wider progress optimism that characterizes the European Enlightenment; human life could be better, should be better and it would get better than it was. This change of view on life was linked to a gradual improvement in living conditions, in particular for the new middle class.

### Doubt that Happiness has Raised

Many changes envisioned by the Enlightened thinkers in the eighteenth century have become true. Reason has largely replaced traditional belief in modern society with its large research industry and expanded educational sector. Rationality has pervaded many life domains, such as health care and management. Political rule is no longer based on heritable rights but has become more democratic and technocratic. As a result, the material standard of living has improved to such a degree that the average citizen lives now more comfortably than kings did in the past. We live not only more comfortably, but also much longer, since life-expectancy at birth has almost doubled over the last 200 years.

Though progress in these fields is undisputed, there is still doubt that we have become any happier and there are even claims that happiness has declined. Such notions are found both among nostalgic romantics, and among hard-core social scientists. The doubts roots both in theory and in empirical indications.

#### Theoretical Conjectures

Several theories of happiness imply that average happiness will remain at the same level in the long run. One such theory is that human happiness is bound to an innate ‘set-point’ (Lykken 1999). In this context Cummins (2010) holds that happiness is maintained homeostatically at around a level of 7.5 on scale 0–10, much like we maintain a body temperature of 36°. In his view we can be less happy when adverse conditions defeat homeostatic corrections, but will not get happier once living conditions are tolerable.

A similar prediction is implied in the theory that happiness depends on comparison and in particular on social comparison. In this view our happiness depends on being better off than your reference group, typically compatriots. Since these profit equally much from social progress, the distance remains the same and so the level of happiness. A variant of this theory holds that happiness depends on the gap between what we want and what we have and that progress is typically accompanied by rising aspirations, which nullifies the effect on happiness (Brickman and Campbell 1971). In this view the pursuit of happiness has set us in a *hedonic treadmill*, on which we run in vain for greater happiness.

Some theories hold that happiness has declined in modern society, because of the negative effects of modernization. In his book *Unbehagen in der Kultur* (Society and its discontents) Freud (1928) holds that happiness lies in indulgence of primitive passions, which is incompatible with the functioning of civilized society and that societal progress has therefore made us less happy rather than more. Likewise several critics of modernization argue that the negative side effects outbalance the positives of rationalization, such as by the attendant alienation and weakening of social bonds. A recent spokesman for this view is Lane (2000) in his book. ‘The loss of happiness in market democracies’.

#### Empirical Indications

Enlightened emphasis on fact based knowledge has resulted in the development of social statistics, part of which involves the systematic logging of miseries, such as theft, murder and poverty. Though many such ills have lessened over time, some negative developments stand out, such as the rise of suicide rates in the late nineteenth century and today’s ‘epidemic of depression’ (e.g. Easterbrook 2003).

Survey research on happiness started in the second half of the twentieth century and the first comparisons over time in a few nations did not reveal any clear trends. Though income per head had doubled in the US, average happiness had remained at the same level. This pattern was first described by Easterlin (1974).

These indications fit the above mentioned theoretical conjectures and together cast serious doubt on the promise of greater happiness embodied in Enlightened progress optimism and even challenge Enlightened optimism as such.

### Plan of this Paper

In the context discussed above I take a closer look at the empirical research on the trend of happiness in nations. Has happiness really remained at the same level? Or has happiness declined? I will first define the concept of happiness in more detail and, based on this, select appropriate indicators. Next I will take stock of the available scores on these indicators in samples taken from the general population in nations. I will then assess the changes in happiness over time in nations and count the cases of changes to the positive and negative. I will also estimate the size of the average change in nations. I will conclude that happiness has risen in most nations. I will close with a discussion of why this conclusion differs from earlier readings of the data and I will also consider what this finding means for the trend in wider quality of life.

## Concept and Measures of Happiness

Most Enlightened thinkers used the term ‘happiness’ in the broad sense of living a good life, to which we refer today using the terms ‘wellbeing’ and ‘quality of life’. Used in this broad sense the word is an umbrella term, which covers several different qualities of life. I have distinguished four different qualities of life elsewhere, on the basis of two distinctions: a difference between *chances* for a good life and actual *outcomes* of life and a distinction between qualities in the *environment* and qualities in *one*-*self* (Veenhoven 2000). Together these two bipartitions provide the four qualities of life presented in Table 1.

**Table 1:**
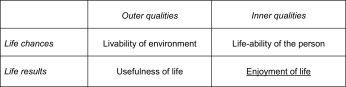
Four qualities of life

*Source*: Veenhoven (2000)

I have argued that three of these qualities cannot be measured comprehensively, but that subjective enjoyment of life (bottom right quadrant in Table 1) is well measurable. I have also argued that overall quality of life cannot be measured, not only because three of the four components cannot be measured comprehensively, but also because there is no sense in summing across quadrants in Table 1. Chances cannot be meaningfully added to outcomes and outcomes and it does not make sense either to sum environmental chances (livability) to inner life-abilities, since it is the *fit* that matters. Consequently quality of life cannot be measured using the usual sum scores, such as the Human Development Index. In my view the most comprehensive indicator of quality of life in a nation is how long and happy people live; this outcome measure indicates indirectly whether the life chances suffice in a country. In this journal I recommended using Happy Life Years (HLY) as an indicator of ‘apparent quality of life’ in nations (Veenhoven 2005).

For these reasons I focus in this paper on happiness in the sense of subjective enjoyment of life. This concept fits the meaning addressed by at least some of the Enlightened thinkers, such as Jeremy Bentham.

### Definition of Happiness

Happiness is the degree to which a person enjoys his or her present life-as-a-whole. In other words, how much the person likes the life he/she leads. ‘Life satisfaction’ is a synonym.

The concept of happiness denotes an *overall* evaluation of life. So the appraisal that life is ‘exciting’ does not mark it as ‘happy’. There may be too much excitement in one’s life, and too little of other qualities. The overall evaluation of life involves all the criteria figuring in the mind of an individual: how good they feel, how well their life meets their expectations, etc. The object of evaluation is *life*-*as*-*a*-*whole*, not a specific domain of life, such as work-life. Enjoyment of work will add to an individual’s the appreciation of life, but does not constitute appreciation of their whole life.

Appraisals of life can concern different periods in time: how life has been, how it is now, and how it will probably be in the future. These evaluations do not coincide necessarily; one may be positive about one’s past life, but negative about the future. The focus of this paper is on satisfaction with present life.

When appraising how much we appreciate the life we live, we draw on two sources of information: (1) how well we feel generally, and (2) how well our life-as-it is compares to standards of how-life-should-be. These sub-appraisals are seen as ‘components’ of happiness, respectively the *affective component* called ‘hedonic level of affect’ and the *cognitive component* called ‘contentment’. This distinction is discussed in more detail in Veenhoven (2009), together with a theory about difference in the determinants of these components.

### Measurement of Happiness

Measurement has long been understood to be an ‘objective’ and ‘external’ assessment, analogous to the measurement of blood pressure by a doctor. By now, we know that happiness cannot be measured this way. Like most attitudinal phenomena, happiness is only partially reflected in behavior. Though some social behaviors tend to be more frequent among the happy, i.e. active, outgoing, friendly, such conduct is also observed among unhappy persons. Likewise, non-verbal behaviors such as frequent smiling or enthusiastic movements appear to be only modestly related to self-report of happiness. Consequently, estimates of someone’s happiness made by peers can be wrong. Suicidal behavior is probably more indicative of happiness. Almost all people who attempt to, or who commit, suicide are unhappy, however, not all the unhappy resort to suicide as a way out of their situation. In fact, only a fraction does.

#### Survey Questions on Happiness

Since it is not possible to measure the happiness of individuals by making inferences based on their overt behavior, we must make do with questioning. That is, simply asking people how satisfied they are with their life-as-a-whole. Such questions can be posed in various contexts, clinical interviews, life-review questionnaires and common survey interviews. The questions can be posed in different ways, directly or indirectly and using by single or multiple items. A common survey question is:

Taking all together, how satisfied or dissatisfied are you currently with your life as a-whole?Next to such questions on ‘overall happiness’, there are also measures specific to the above mentioned ‘components’ of happiness. Hedonic level of affect can also be measured using affect balance scales and multiple moment assessment, such as the Day Reconstruction Method. Trend data on hedonic level in nations is scarce as yet and is therefore not used in this analysis. There is more trend data on contentment as measured with Cantril’s (1965) ladder rating and these data are included.

#### Doubts About Self Reports

There are many qualms about simple self-reporting of happiness, in particular about its validity and the comparability off the answers across nations. Elsewhere I have considered the objections and inspected the empirical evidence for claims about bias (Veenhoven 1993). I found no proof for any of the objections, so I assume that happiness can be measured in this way. Others have come to the same conclusion (Diener 1994; Saris et al. 1998). Suffice to note that cross-national differences in happiness correspond in the expected way with rates of depression across nations (Van Hemert et al. 2002), and suicide (r = −.46).

## Data on Happiness in Nations

### World Database of Happiness

Data on average happiness in nations is available in the World Database of Happiness (Veenhoven 2012a). This is a ‘findings archive’ in which the results of empirical research are gathered that are yielded with measures that fit the concept of happiness as life-satisfaction. All acceptable indicators are included in the collection ‘Measures of Happiness’ (Veenhoven 2012b).

Most measures are single survey questions, such as mentioned above. This is just one of many acceptable measures of happiness. Survey questions have used different key words, such as ‘happiness’, and different response options, such as verbal scales. Next to these single questions there are also multiple questions, some of which constitute a ‘balance scale’.

This diversity of measures of happiness used in the many surveys makes it difficult to compare scores and in particular to assess change in average happiness over time. Therefore the World Database of Happiness sorts the different measures of happiness into ‘equivalent’ kinds, that is, into questions that address happiness using the same keyword and a rating scale of the same length.

Research findings yielded using these acceptable measures of happiness are described in standard excerpts using standard terminology. Two kinds of findings are distinguished, ‘distributional findings’ and ‘correlational findings’. Distributional findings denote how happy people are in a particular population and are often summarized in a measure of central tendency, typically the mean. Correlational findings are about things that go together with more or less happiness and summarized using measures of association, such as Pearson’s correlation coefficient.

Distributional findings are sorted into findings among special publics, such as elderly persons, and findings in the general population. The findings on happiness in the general public are further subdivided by the kind of areas from which samples were drawn, such as ‘regions’, ‘cities’ and ‘nations’. These latter findings are gathered in the collection of ‘Happiness in Nations’ (Veenhoven 2012c), which I use for this research.

### Collection Happiness in Nations

To date (November 2013) the collection ‘Happiness in Nations’ contains 5568 findings on average happiness of the general population in 164 nations over the years 1946–2012. These findings are sorted into three levels, one by nation, two within nations by kind of measure used and three within measures of the same kind by year.

An example of a ‘nation page’ is presented in “Appendix”. This is the case of Brazil for which 37 distributional findings in the general public are available. These findings are sorted into blocks of equivalent survey questions. The first block of question type 111b has only one finding in the year 1975 and therefore provides no information about change over time. The second block consists of 6 findings yielded by a survey question on how ‘happy’ one is, the answers to which were rated on a 4 step verbal response scale. The measure codes link to the precise text of that question and detailed information about the investigation can be found behind the ‘i’ icon.

Findings are sorted by year within each block, and this second block consists of six findings the years 1990, 1997, 2002, 2003, 2006 and 2008. Looking at the blocks in “Appendix”, we see no clear trend in the responses to the question on happiness (measure type 111c) between 1990 and 2008, but a positive trend in the responses to questions about life-satisfaction (measure type 121C and 122F/G) and ratings on the Cantril ladder (measure type 31D). All series depict ups and downs.

### Selection of Time-Series

Using this collection of Happiness in Nations, I gathered time series of average happiness in nations that are based on (1) identical survey questions and (2) cover a period of at least 10 years. I did this together with Floris Vergunst in the context of an analysis of the relation between happiness and economic growth in nations (Veenhoven and Vergunst 2014). The full data matrix is reported in this paper.

#### Identical Questions

Within the blocks of equivalent questions discussed above, there are still small differences in the wording of the lead question and/or response options. These variations are marked by the last symbol in the measure code. There are also variations in the timeframe addressed in the question, and these are marked with the third letter code, where ‘c’ stand for ‘current’, ‘g’ for in ‘general’ and ‘u’ is used for ‘unclear’. These minor variations in the wording of questions can result in small differences in the mean scores and could as such overshadow the small changes in actual happiness over time. For that reason we limited our data set to time-series based on identical questions, that is, questions with the same measure code.

In the above mentioned case of six questions on how ‘happy’ one is in Brazil this meant that we considered only the three findings based on the question variant ‘a’, which now show an upward trend.

#### Series of Average Responses

On this basis we constructed several series of responses to identical questions on happiness in the same nation over time. Since we focus on the long-term, we limited our analysis to series that covered a minimum of 10 years. We also limited the analysis to data gathered using probability samples. If the same question was used in several surveys in the same year in the same country, we used the average response to that question, e.g. in the case of the Eurobarometer surveys, which are held twice a year in European member states we took the average of these two observations. We did not require that a series involved more than two data points, though most series involve more.

This resulted in 199 time-series for average happiness in 67 nations, which together gave 1531 data points. For detail see Veenhoven and Vergunst (2014).

## Analysis

The question at stake is whether average happiness has typically remained at the same level, or has risen in most nations. To answer this question we first assessed change in each of the 199 series of responses to the same question on happiness in the same country. Next we computed the average change over all series per country.

### Change of Average Happiness in Series of Identical Questions

We regressed happiness against year for all the 199 time series using. The resulting linear regression coefficients were used to indicate the yearly change in happiness in the period covered by the series. Since happiness is expressed on range 0–10, a regression coefficient of 0.01 means a rise of 0.1 point per year, which amounts to a 1 point gain in happiness over 10 years. These yearly coefficients were used in the following ways.

#### Ratio of Rise or Decline

We first counted the number of series in which happiness had gone up in a country and the number in which happiness had gone down. Then we assessed the ratio of rise and decline; a ratio >1 would indicate that increasing happiness was more common than decline; a ratio of 1 that rising and declining happiness were equally frequent, and a ratio smaller than one would mean that a decline in happiness was the most common trend.

#### Average Change Coefficient

The above bi-partitions provide a view of the relative frequency of rise and decline in happiness in nations, but do so at the cost of loss of variation. In order to use the available variance more fully we computed the average change in happiness over all 199 series and assessed whether that average coefficient was positive or negative.

### Change of Average Happiness in Countries

Using the change coefficients in the series, we computed the average change coefficients for each of the 67 nations. Where only one series was available, we took the change coefficient observed for that one and when more series were available we computed the average change score.

These change scores in nations were analyzed in the same way as the change scores in the series. First a ratio of rise or decline in happiness was obtained and then the average change scores were computed and we assessed the statistical significance of these scores.

## Results

Of the 199 series 67 % showed a rise in happiness and 33 % a decline, which resulted in a ratio of 2.0. Likewise happiness rose in 62 % of the 66 nations and declined in 38 %, which is a ratio of 1.6. See Table 2. This is clearly more than the ratio of about 1 that would denote that happiness remained unchanged over the ups and down over time.

**Table 2:** Change of average happiness in nations 1950–2010 Frequency of rise versus decline

Pattern of change	Series		Nations	
	*N*	*%*	*N*	*%*
Rise	133	67	41	62
Decline	66	33	25	38
Total	199	100	66	100
Ratio rise-decline	2.02		1.63	

*Source*: Veenhoven and Vergunst (2014)

The average yearly rise in happiness observed in the 199 series was +0.016. The average rise in the 67 nations was +0.012.

These numbers may seem small at first sight, but they result in a considerable improvement in happiness in the long term. At the growth rate of 0.012, average happiness will rise one point on a 0–10 scale in 83 years. Given that the actual range on this scale is between 2.5 and 8.5 (Veenhoven 2012d), a one point rise equals a gain of 17 %.

Is the growth in happiness observed here really part of a longer trend? Remember that we considered time-series of at least 10 years. We can see from Table 3 that the average change in happiness does not differ very much between the long and very-long term and that the rise is slightly stronger in the longest term, that is, 40 years of more. So the gradual growth of happiness has been fairly continuous during this 1950–2010 period.

**Table 3:** Change of average happiness in nations Average yearly change in points on scale 0–10, split-up by length of period

Period	Series		Nations	
	*N*	*b*	*N*	*b*
10–20 years	114	+0.017	31	+0.010
20–40 years	67	+0.013	27	+0.009
>40 years	18	+0.020	9	+0.030
Total	199	+0.016	67	+0.012

*Source*: Veenhoven and Vergunst (2014)

## Discussion

So the Enlightened thinkers were right. Happiness is apparently possible during our earthly life, average happiness in developed nations is about 7.5 on scale 0–10 and is now 8.3 in contemporary Denmark. Moreover the analysis reported in this paper shows that average happiness has risen in most nations since the first measurements in the 1950s.

This result gives rise to three questions, one why do so many learned people think than happiness declines, and two, whether this rise in subjective appreciation of life also denotes a rise in wider quality of life. The third question is how much we have improved since the days of the Enlightenment.

### Why do These Results Differ from Earlier Analyses of the Trend in Happiness?

The first surveys of happiness in developed nations left no doubt that most of the people living there were happy, but it took some time before changes in average happiness became visible. One reason is that the long-term trend is disguised by short-term variations caused by events, such as ups and downs in the economy. Another reason is in inaccuracies of measurement, such as those due to the variations in the place of a question in a questionnaire, differences in wording of survey questions and variations in sampling. To see the long-term trend through this dust, we need a lot of observations and with its 1531 data points this analysis used more observations than any previous study. The law of greater numbers helped us to see a general pattern.

Another thing that has plagued earlier analyses is that they have focused on a few particular countries and generalized the results to the rest of the world. The case of the USA is presented as a typical case, while the stagnating happiness seen in this country is an exception, not the rule. The same holds for Japan, where the view on the trend is moreover clouded by changes to the wording of the survey questions on happiness (Suzuki 2009).

### Does Rising Happiness Denote a Better Quality of Life?

Most Enlightened thinkers thought of more than just greater satisfaction with life and had wider qualities of life in mind. The observed rise in this outcome of life (right bottom quadrant in Table 1) does suggest that life chances (top quadrants in Table 1) have also improved. There is indeed massive evidence of great improvements in living conditions (top-left quadrant) in particular in the material standard of living See e.g. Madison (2007). Likewise life-abilities (top right quadrant) have improved much, among other things as the result of better education.

### How much Happier than at the Time of the Enlightenment?

Since empirical happiness research started in the 1950s, we do not know how happy Europeans were in the eighteenth century. Still we do know a lot about living conditions at that time, such as the income per head and the frequency of homicide. For these conditions we know how they relate to average happiness in contemporary societies; see e.g. the review article by Dolan et al. (2008) and the earlier mentioned World Database of Happiness. On that basis it is no wild guess that average happiness was much lower in Europe and probably around 5 on the 0–10 scale, as we see today in Pakistan (Veenhoven 2012d).

The average in Western-Europe is now around 7.5, which is some 2.5 point higher. Extrapolation of the rise in happiness observed over the last 60 years fits that estimate. The average yearly rise in the 67 nations observed here was +0.012 on scale 0–10, which equals a rise of one full point every 83 years. At this rate happiness must have improved by more than two points over the past two centuries and must thus have been around 5.

This rise in happiness is paralleled by improvements of health and longevity, which also reflect human flourishing. The average length of life has almost doubled in most nations. As a result w now live longer and happier than ever before in human history, which means that the chances provided by this kind of society fit human nature very well.

This is not to say that the ‘Enlightenment project’ does not involve any disadvantages, but these are apparently outbalanced by the many advantages of guidance by reason. For a more detailed discussion of this point see my earlier article in this journal (Veenhoven 2010).

## Conclusion

The newly available data refute earlier claims that we did not get any happier. Average happiness has risen in most developed nations over the last decade and is now probably much higher than in the days of the European Enlightenment. The perspective that we can create greater happiness for a greater number has born fruit.
